# Effects of medial-lateral destabilisation insole on neuromuscular control strategy during weight-bearing tasks in people with chronic ankle instability: a randomised crossover laboratory trial

**DOI:** 10.1186/s13102-026-01585-0

**Published:** 2026-02-11

**Authors:** Zongchen Hou, Ting Zhu, Shuang Ren, Xin Miao, Si Zhang, Dong Jiang

**Affiliations:** Department of Sports Medicine, Beijing Key Laboratory of Sports Injuries, Engineering Research Centre of Sports Trauma Treatment Technology and Devices, Peking University Third Hospital. Institute of Sports Medicine of Peking University, Ministry of Education, Beijing, 100191 China

**Keywords:** Chronic ankle instability, Unstable footwear, Neuromuscular control, Rehabilitation

## Abstract

**Background:**

Impaired neuromuscular control is a feature for people with chronic ankle instability and could be restored by the destabilising footwear, yet there is a lack of destabilisation insole design targeting medial-lateral neuromuscular control. This study investigated whether a medial-lateral destabilising insole (medial-lateral density gradient + heel lift) acutely enhances peroneus longus (PL) activation, ankle inversion control during gait, and postural stability during single-leg standing in CAI.

**Methods:**

The study followed the randomised crossover laboratory design. Twenty CAI participants and twenty healthy controls performed walking trials at self-selected speeds and eyes-closed single-leg standing under two conditions (destabilising vs. flat insole) randomly. Joint angle, electromyography of lower extremity during gait and centre-of-pressure (COP) parameters during stance were analysed using two-way repeated statistical parametric mapping and ANOVA.

**Results:**

Compared to the flat insole, the destabilising insole significantly increased ankle inversion angle (mean differences = 2.7°; −56 to + 100 milliseconds relative to heel strike; *P* = 0.041) and normalised PL activation (mean differences = 20.1%; −100 to + 80 milliseconds; *P* < 0.001) exclusively in the CAI group during gait. During standing, destabilising insoles reduced time-to-boundary minima in the medial-lateral direction (η² = 0.487; *P* < 0.001) in CAI and increased medial-lateral (η² = 0.213, *P* = 0.036) and anterior-posterior COP velocity (η² = 0.170, *P* = 0.012) in both groups.

**Conclusion:**

The destabilising insole acutely enhanced ankle inversion control and PL activity during gait in CAI individuals while challenging postural stability during standing for healthy and CAI individuals. These findings support its potential as a supervised intervention to augment sensorimotor training during weight-bearing tasks.

**Trial registration:**

Chinese Clinical Trial Registration, ChiCTR2100046146. May 5^th^ 2021.

## Introduction

Ankle sprains are among the most prevalent injuries in daily life and sports [1]. Approximately 40% of people developed recurrent ankle sprains after the first initial injury, termed chronic ankle instability (CAI) [2]. CAI is associated with multiple impairments, including pathomechanical, sensory-perceptual, and motor-behavioural impairments [3]. Within motor-behavioural impairments, altered neuromuscular control is a key feature [4–6]. Past research has shown that CAI participants had insufficient peroneal activation, increased ankle inversion angles during ground contact phase of walking [4, 5] and decreased postural control stability during standing [6], which were thought to be associated with heightened re-injury risk in this population.

To address the neuromuscular alternations, numerous conservative treatment methods including manual therapy, kinesiology tape, proprioception and strength training has been developed [7]. Among them, destabilising footwears have been developed as a convenient tool to challenge dynamic stability during movements. These interventions typically induce incorporates instability structure via modifying the midsole [8] or outsole [9, 10] of the shoes. Among them, rocker shoes such as (Masai Barefoot Technology (MBT)) are the most widely used and primarily provide instability in the anterior–posterior direction [9]. However, their ability to perturb medial–lateral postural control is limited, despite deficits in this plane being more pronounced in individuals with CAI [11]. To specifically target medial–lateral instability, some prototype footwear designs have incorporated articulating mechanisms beneath the sole [12] to induce medial-lateral destabilisation. Although these footwear designs showed potential to enhance neuromuscular activation, the high manufacturing cost (approximately $475 for a pair) [13] and potential safety concern (excessive inversion movement around 45 degrees) [13] limited its application in the practice.

Insole is another accessible and cost-effective biomechanical intervention to alter the neuromuscular control around the ankle joint. The lateral wedge is the most common insole type with the objective of everting the subtalar joint for a proper joint alignment. Past research has shown that the lateral wedge could decreased ankle eversion and dorsiflexion angles [14, 15] during gait in the healthy population and increased facilitates muscle reaction speed [16] and postural stability during stance [17] in people with CAI, potentially fulfilled the preventive purpose in the management of CAI. However, the lateral wedge insole cannot provide proper instability stimuli to ankle joint in the process of enhancing neuromuscular control in the unstable environment [18]. Medial wedge insole was originally designed to promote ankle supernation movements for people with pronated foot [19, 20]. The medial-perturbation feature makes it potentially reasonable to facilitate neuromuscular control compared to tradition preventive insole. However, the effect of such mediolateral perturbation insoles on kinematics and EMG during walking and postural stability during single leg standing in CAI population remains unclear.

To address the shortcomings of safety, high expenses and lack of rehabilitative function in the previous footwear, this study investigated a custom destabilisation insole incorporating a heel lift and dual-density materials (softer lateral, stiffer medial), which induced reasonable inversion perturbation (fifteen degrees during standing) with a pair expense of $58. We further examined its acute effects on kinematics and EMG during walking and postural stability during single leg standing in individuals with CAI and healthy controls. We hypothesised that, compared to a flat insole, the destabilisation insole would: (1) increase peroneus longus amplitude and ankle inversion angle at initial contact during gait in both groups, and (2) decrease postural stability (e.g. time to boundary and centre of pressure excursion velocity) during standing in both groups.

## Methods

### Design

The study followed randomised crossover laboratory study design adheres to CONSORT guidelines. The sample size was calculated by normalised peroneal longus muscle amplitude differences between the instability footwear (0.48 ± 0.18 maximum voluntary isometric contraction (MVIC)) and shod condition (0.25 ± 0.15 MVIC) during gait in a similar study [12]. The calculated effect size was 1.38. By setting the level of significance to 0.05 and the statistical power to 0.95 in a two-tailed paired-t test, the estimated required sample size was 10 with the actual power of 0.97. The independent variables were group (CAI and control) and insole condition (new insole and flat insole). The outcomes were evaluated by the postural control during single leg standing and electromyography (EMG) and kinematics during gait. EMG data were collected and analysed in both groups for the anterior tibialis, fibularis longus, lateral gastrocnemius, rectus femoris, biceps femoris, and gluteus medius.

### Participants

Twenty individuals with self-reported CAI (mean age, 28.9 ± 3.3 years) and twenty healthy individuals (mean age, 27.8 ± 5.6 years) completed the study **(**Table 1). The inclusion criteria for CAI [21] were (1) first ankle sprain occurred 1 year ago, (2) no ankle sprain within the past 6 weeks, (3) Cumberland Ankle Instability Tool (CAIT) [22] scoring < 24, Foot and Ankle Ability Measure [23] on the activity of daily life subscale (FAAM-ADL) scoring < 90%, FAAM-Sports scoring < 80%, and (4) at least 2 episodes of “giving way” or “instability” during daily activities or sport activities within the past 6 months. (5) considering that the foot posture may influence gait biomechanical response with insoles [24], only participants with neutral foot (foot posture index, FPI = 0–5) were included. Any participants with bilateral injury, a history of lower extremity surgery or any illness that might have influenced experiment were excluded.

**Table 1:** Demographics of participants

	CAI (*n* = 20)	Control (*n* = 20)
Age	28.9 ± 3.3	27.8 ± 5.6
Number	9 M, 11 F	10 M, 10 F
BMI (kg/m^2^)	22.3 ± 3.3	21.1 ± 4.1
FAAM ADL (%)	85.9 ± 9.2	100
FAAM SPORT (%)	70.9 ± 7.6	100
CAIT	10.8 ± 3.7	27.9 ± 1.9
FPI	3.3 ± 1.4	2.9 ± 2.0

*BMI* body mass index, *FAAM ADL/SPORT* Activities of daily life/Sport subscale of Foot and Ankle Ability Measure, *CAIT* Cumberland Ankle Instability Tool, *FPI* Foot posture index

Specific inclusion criteria for the control group include (i) no history of ankle sprain injury in their lifetime, (ii) a score of 100% on the FAAM-ADL and FAAM-Sports, (iii) CAIT questionnaires scoring 30. Written informed consent was obtained from all participants in accordance with the Declaration of Helsinki, as approved by the Research Ethics Committee of Peking University Third Hospital (IRB00006761-M2024043) before participation.

### Instruments

**Fig. 1:**
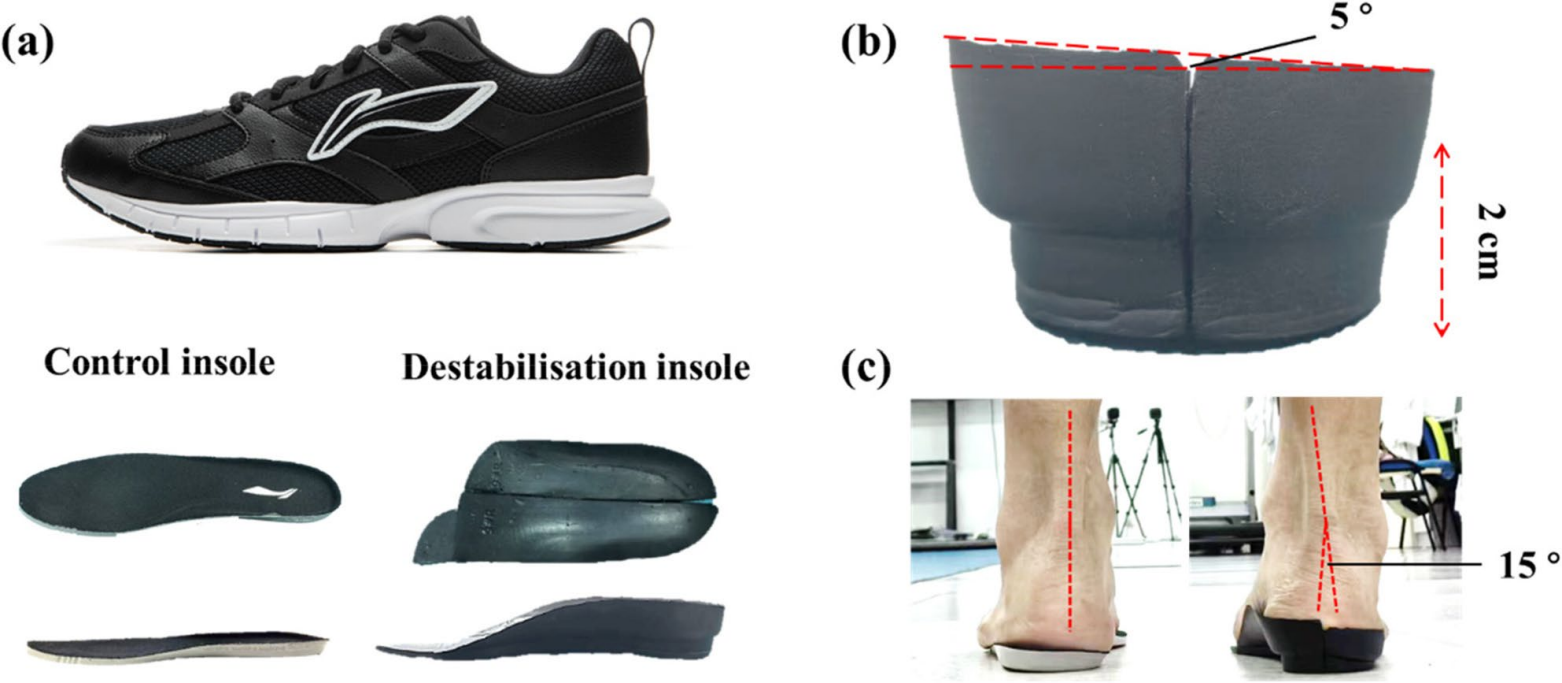
Demonstration of standardised sports shoes and insoles details. (**a**). Lateral and top view of shoes and different insoles used in this study. (**b**). Back view of destabilisation insole without weight. The insole has natural inclination around 5 degrees and heel height around 2 centimetres. (**c**). Back view of different insoles with static double-limb standing position for a participant in the control group (left side on the normal insole and right side on the destabilisation insole). The destabilisation insole has a 15-degree inversion inclination under loading while the control insole has a neutral position under loading

EMG data was measured with a DELSYS wireless EMG sensor (Trigno™ Wireless Systems, Delsys Inc., USA) sampled and synchronised through the Vicon system at 2000 Hz. Three-dimensional trajectories of the reflective markers were collected using a twelve-camera motion capture system (VICON, Oxford, UK) at a sample rate of 100 Hz. Ground-reaction forces (GRF) were collected using two embedded force plates (AMTI, Watertown, MA, USA) at a sample rate of 2000 Hz. To minimise the effects of hardness of shoes on the neuromuscular performance [25], standardised regular sports shoes (ARSU067-1, Lining, China) were used in both insole conditions (Fig. 1a).

### Ankle destabilisation insole

The custom medial–lateral destabilisation insole was a three-quarter length design, extending from the heel to the metatarsal heads. It was constructed with a dual-density ethylene-vinyl acetate (EVA) foam core. The medial aspect of the rearfoot and midfoot was fabricated from a higher-density material (50 Shore A), while the lateral aspect utilised a lower-density material (30 Shore A). This gradient created a built-in frontal-plane inclination. Under full body weight during static standing, the insole produced a 15° of ankle inversion, measured as the angle between the rearfoot vertical axis and the ground (Fig. 1c). Additionally, the insole incorporated a 20-mm heel lift, which maintained the ankle in approximately 7° of plantarflexion relative to the neutral shod condition. The combined design—medial density gradient, inversion inclination, and heel lift—aimed to place the ankle in an inverted and plantarflexed posture at initial contact during gait. This posture was intended to provide a controlled medial-lateral perturbation, thereby increasing the demand on the peroneal musculature for stabilisation.

### Testing procedures

All participants were allocated with control insole and destabilisation insole in the randomised order (https://www.randomizer.org/). Given the destabilisation design of the insole, the participants and researcher who conducted the testing were not blinded from the orders of insole style. The statistical technician was blinded with group allocation. In each insole condition (control or destabilisation insole), all participants were required to perform the warm-up, single leg standing and walking task in the fixed order. Each participant was provided with 5 min rest to avoid any learning effects between footwear condition.

#### Warm-up

Participants completed an informed-consent form and a health questionnaire to determine their eligibility for inclusion in the study. Then all participants were allocated with control insole or destabilisation insole allowed to walk on the treadmill with 4 km/h until they felt comfortable with the current insole condition. No participants practised for more than 5 min to avoid any potential muscle fatigue.

#### Walking

 Subjects performed three walking trials in a self-selected speed over the force plate (AMTI, Watertown, MA, USA) with 2000 Hz sampling rate. Ground contact was defined as the moment over 15 N. Joint kinematics and EMG were collected during 200ms before and after the ground contact. For the joint kinematics measurement, A total of 22 reflective markers (9.5 mm in diameter) was fixed to the anterior superior iliac spine, posterior superior iliac spine, greater trochanter, lateral epicondyle, medial epicondyle, lateral malleolus, medial malleolus, first metatarsal head, fifth metatarsal head, toe, and heel. For the EMG measurement, the skin was shaved and cleaned with a 70% alcohol solution to ensure the correct conductivity of the myoelectrical pulse. Locations of EMG electrodes were determined according to the SENIAM [26]. The EMG signals were measured for the following muscles during walking: gastrocnemius lateral (GL), tibialis anterior (TA), rectus femoris (RF), biceps femoris (BF), gluteus medius (GM), peroneal longus (PL). Only the injury side data in the CAI group and left side data in the healthy group was included into the analysis.

### Maximum voluntary isometric contraction procedures

Following the testing procedure, all participant performed 3 progressive submaximal trials of 5 s in duration to familiarise the testing procedure. This was followed by one 5-second Maximum voluntary isometric contraction (MVIC) trial. All resistance was applied by a chair with a strap belt. The positioning of the participant and investigator used for the ankle [27], knee [28] and hip [29] musculature followed the methods proposed before except for the lateral gastrocnemius, where the participant was tested in a standing position with the knee in full extension.

### Data processing

#### Joint kinematics

 The joint kinematics and EMG processing procedure were reported before [29]. The initial contact was defined as the first frame with vertical ground reaction force over 15 N. The interest period was chosen from 200 ms before to 200 ms after initial contact. Kinematic markers trajectories were filtered using a fourth-order low-pass Butterworth filter and a 10-Hz cut-off frequency. Ankle, knee, and hip joint angles were computed with a Euler X-Y-Z order of rotations [i.e., flexion (+) and extension (−), adduction (+) and abduction (−), and internal rotation (+) and external rotation (−)].

#### EMG

 Raw EMG signals were processed offline using a standardised protocol [30]. First, signals were visually inspected, and any segments with obvious artifacts (e.g., movement artifacts or baseline shifts) were excluded. The data were then band-pass filtered (20–450 Hz) using a zero-lag, fourth-order Butterworth filter to remove low-frequency movement artifact and high-frequency noise. The filtered signals were full wave rectified. To obtain a linear envelope representing muscle activation amplitude, the rectified signals were smoothed using a root-mean-square (RMS) algorithm with a 100-ms moving window. For normalisation, the mean RMS value identified during the MVIC trial was used as a reference. The RMS amplitudes from the gait trials were subsequently expressed as a percentage of this mean MVIC value (%MVIC).

### Data analysis

Shapiro‒Wilk tests were used to assess the normality of data. All statistical parametric mapping (SPM) analyses were implemented using the open-access SPM1D code (www.spm1d.org) in MATLAB R2019a (The MathWorks, MA, USA). To compare joint angle and EMG differences in different conditions across groups, two-way repeated SPM was applied in the analysis. If any interaction was observed, paired-t SPM was applied to calculate the maximum difference between conditions within group. The postural control measurements were analysed with two-way repeated ANOVA in SPSS software 16.0 (IBM Corporation). If any interaction was observed, post-hoc analysis with Bonferroni correction was conducted to calculate the condition differences within group. Partial eta squared (η^2^) was used to indicate the effect size of the two-way ANOVA’s interactions and main effects with the thresholds: 0.01∼0.06 for small, 0.06∼0.14 for moderate, and > 0.14 for large effect size [31]. Alpha level was set a priori at *p* < 0.05.

## Results

### Lower extremity joint angle in different conditions across groups

**Fig. 2:**
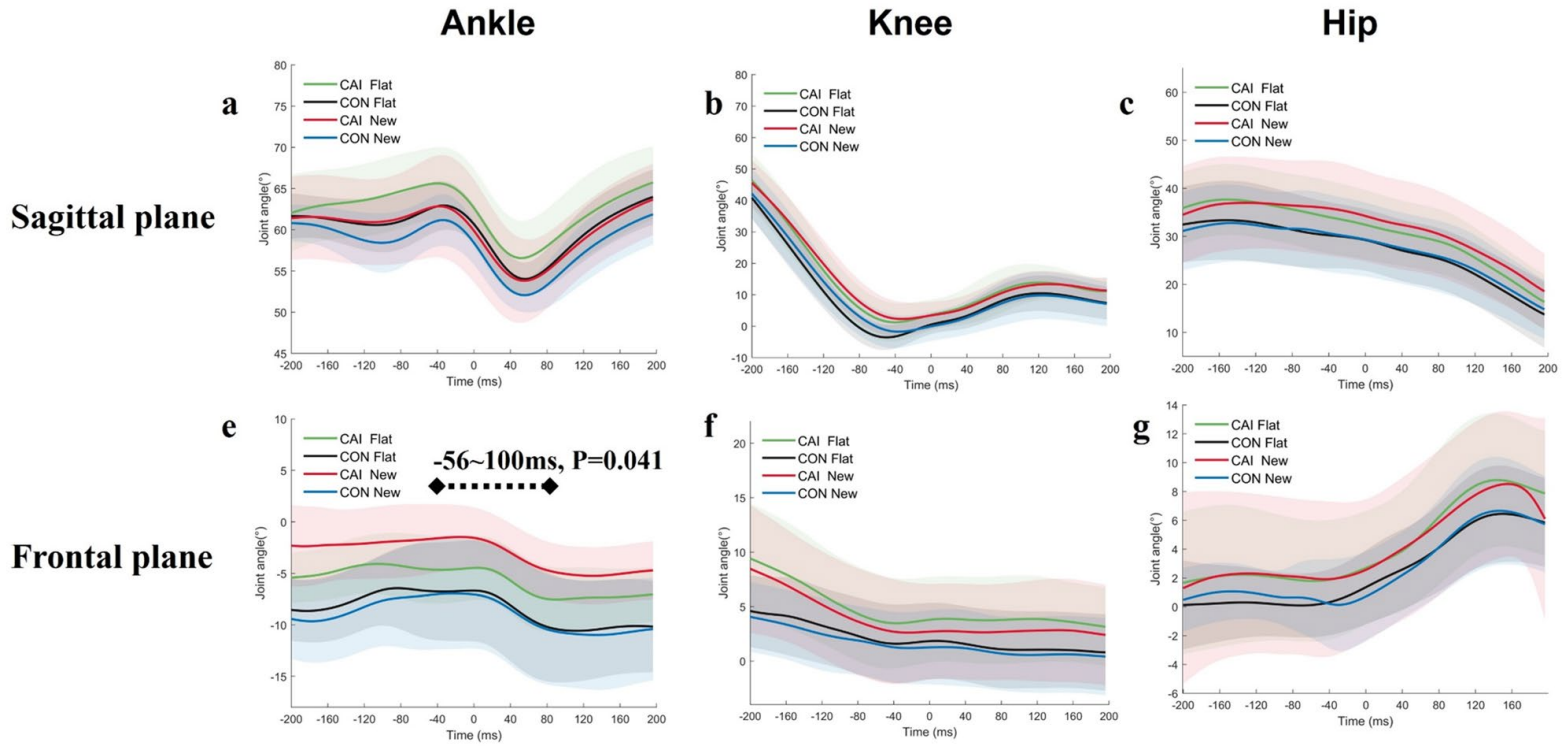
Sagittal and frontal Joint angle comparison in the sagittal (**a**-**c**) and frontal plane (**d**-**f**) between insole conditions across groups

A group x condition interaction on frontal ankle joint angle (*P* < 0.001, F = 7.99) was observed between − 56ms to + 100ms (- indicates prior to initial contact and + indicates after initial contact) during walking. New insole condition demonstrated more inversion than flat insole condition (max differences, 2.7°, *P* = 0.041) from − 56ms to 100ms in the CAI group while remained at the similar level in the control group **(**Fig. 2d**)**. No other differences were found between conditions across groups.

### Lower extremity EMG in different conditions across groups

**Fig. 3:**
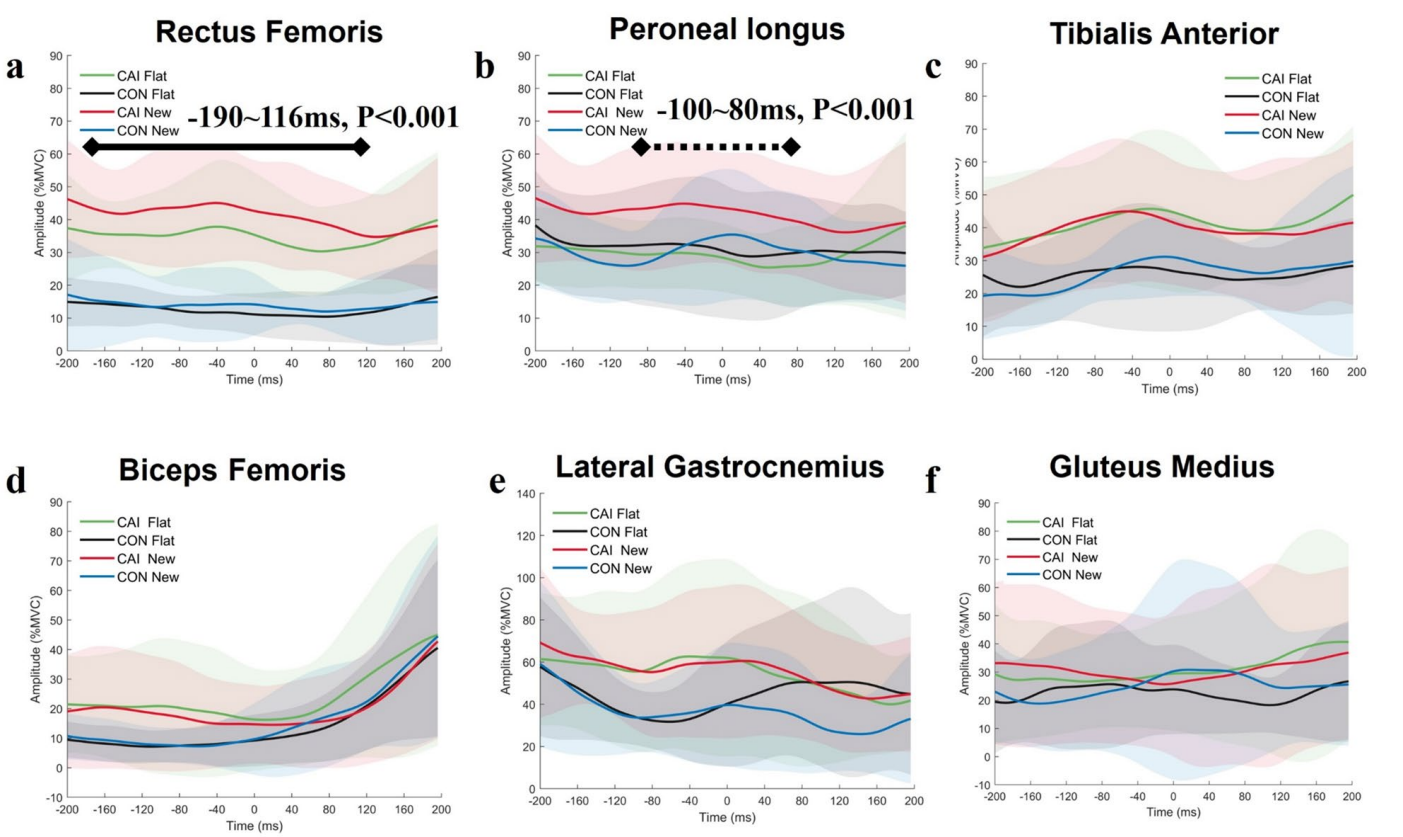
Muscle amplitude of lower extremity comparison between insole conditions across groups. **a**, Rectus Femoris, **b**, Peroneal Longus, **c**, Tibialis Anterior, **d**, Biceps Femoris, **e**, Lateral Gastrocnemius, **f**, Gluteus Medius

A group x condition interaction on normalised peroneal longus (PL) amplitude (*P* < 0.001, F = 9.21) was found between − 100ms to + 80ms during walking. New insole increased PL (max differences, 20.1% MVIC, *P* < 0.001) than flat insole from − 100ms to + 80ms during walking for participants with CAI (Fig. 3b). Significant group effects (*P* < 0.001, F = 6.24) were observed for the normalised rectus femoris (RF) amplitude. Participants with CAI demonstrated higher RF amplitude than the control group (max difference, 26.1% MVIC, *P* < 0.001) from − 200ms to + 118ms during walking in both insole conditions (Fig. 3a).

### Postural control in different conditions across groups

As shown in Table 2, a group x condition interaction effect (*P* < 0.001, η^2^ = 0.487) was observed on the TTB ML mean. When standing with the new insole, CAI group had a decreased TTB ML mean compared with the flat insole while the control group had a similar value between insole conditions. In addition, both groups showed similar decreased TTB AP mean (*P* = 0.012, η^2^ = 0.170) and increased COP ML velocity (*P* = 0.036, η^2^ = 0.213) in the new insole compared to flat insole. Furthermore, CAI group demonstrated higher COP ML velocity (*P* = 0.021, η^2^ = 0.146) across conditions.

**Table 2:** Postural control comparison during single leg standing

**Variables**	**Group**				**P inter (Effect size)**	**P group (Effect size)**	**P shoe (Effect size)**
	**CAI**		**Control**				
	**Flat**	**New**	**Flat**	**New**			
TTB (s)							
ML min	0.088±0.042	0.097±0.050	0.156±0.120	0.144±0.152	0.400(0.021)	0.054(0.105)	0.922(0.001)
ML mean	2.677±0.665	1.942±0.634	2.525±0.698	2.283±0.567	**<0.001(0.487)**	0.644(0.006)	**0.007(0.195)**
ML SD	3.352±0.929	3.272±1.246	3.353±1.582	3.194±1.350	0.530(0.012)	0.221(0.044)	0.542(0.002)
AP min	0.325±0.179	0.316±0.210	0.375±0.176	0.326±0.169	0.649(0.006)	0.522(0.012)	0.510(0.013
AP mean	4.233±0.984	4.099±1.497	3.975±1.153	3.252±0.793	0.088(0.083)	0.142(0.062)	**0.012(0.170)**
AP SD	4.163±0.881	4.286±1.156	3.739±1.198	3.351±1.245	0.509(0.013)	0.060(0.100)	0.206(0.047)
COPV(cm/s)							
ML	4.980±1.596	5.910±2.407	3.659±1.207	5.195±2.713	0.596(0.008	**0.021(0.146)**	**0.036(0.213)**
AP	4.534±2.616	4.485±2.097	3.122±2.601	4.181±2.416	0.356(0.025	0.167(0.056)	0.312(0.030)

Data were expressed in mean ± standard deviation

*TTB * time to boundary, *COPV* centre of pressure excursion velocity, *ML/AP min/mean/SD* minimum/mean/standard deviation in the medial-lateral/anterior posteriordirection

*P*<0.05 was shown in bold font

## Discussion

To our knowledge, this study is the first to examine the acute effects of medial-lateral destabilisation insole among people with CAI and healthy controls. The results revealed that the new insole caused increased ankle inversion angle and peroneal longus activity exclusively in CAI during gait. Furthermore, the new insole significantly increased anterior-posterior and medial-lateral postural instability for both groups. Collectively, these findings indicate that the destabilisation insole acutely challenged postural stability in CAI and healthy individuals and potentially facilitating neuromuscular adaptation in CAI during functional tasks. This supports its potential as a supervised rehabilitation tool for enhancing sensorimotor control during daily weight-bearing activities. 

The primary finding of the study was that the destabilisation insole induced more inversion movements around ankle joint during the heel strike phase during walking. Previous studies have reported that various shoe modifications influence the ankle joint kinematics. For example, the rocker shape sole shoe (Massai Barefoot Technology) reduced hip peak flexion angle and increased knee peak flexion angle [32]. The other shoe attaching with two destabilisation hemisphere elements in heel and forefoot zone to exert perturbations [33, 34] and found that the location (lateral forefoot)^11^ and height (from 9.2 mm to 10.8 mm) [34] of the hemisphere elements induced greater ankle inversion movements around 5° than control shod condition during walking. Our study implemented a simple and convenient insole deign achieving around 2.7° more ankle inversion from pre-contact 56ms to post-contact 100ms. An ankle inversion angle of 14° at foot strike has been reported to incite an event of ankle inversion sprain due to medially deviated ground reaction force and subsequently an inversion moment arm along the ankle joint centre [35]. The current study showed the developed destabilisation insole may function as a safer ankle inversion perturbation orthotics to provide the stability challenge within controllable range. Although the peak inversion angle with new insole (minus 2.12 degrees) during gait was far below the injury threshold, the increased inversion movements might pose potential threat for people with CAI. More longitudinal studies are needed to explore the detailed safety performance of the new insole.

The study also demonstrated significant increases peroneal longus amplitude than flat insole during heel contact phase in the CAI population. This increase in the PL was likely initiated to counteract the frontal instability induced by the inverted and plantarflexed insole shape, which was thought as the primary muscle to resist the ankle inversion movement [9]. The increased fibularis longus amplitude may not only stabilise the joint but also shift the centre of pressure more medially to prevent further injury [10]. Except the EMG changes in the distal (ankle joint), our study also observed an increased rectus femoris amplitude in people with CAI compared to control group in both insole conditions. The EMG amplitude changes around proximal joint (knee joint) could be due to the abnormal neuromuscular control strategy documented in the population with CAI [36]. It was reported that CAI demonstrated abnormal submaximal strength and accuracy of the knee extensor muscles in comparison with controls [36]. To cope with the loading on the lower limb during initial contact, the abnormal proximal neuromuscular control may lead to a greater amplitude of knee muscle to compensate for the instability challenge around ankle joint. However, no other coincide proximal joint kinematics and EMG differences were observed, which was contradict to previous findings with increased knee flexion angle in the CAI [37]. The proximal strategy influenced by the destabilisation insole still needs further elucidation with more experiments.

This study found that CAI group had greater medial-lateral COP excursion velocity during standing compared to the controls under both insole conditions, indicating impaired postural stability irrespective of insole type. This finding aligns with previous reports of medial-lateral postural deficits in CAI during single leg standing whether eyes-open or eyes closed [38, 39]. Notably, our study firstly found that the unstable insole exaggerates the medial-lateral postural instability in the CAI group, demonstrated by greater decrease in medial-lateral time to boundary when switching from flat insole to destabilisation insole. This finding may result from the insole’s combined lateral-deviation [12] and elevated heel [40] design, which contributes to imposing the ankle joint into an unstable position. The lateral-deviation of COP was considered as classic biomechanical characteristics in CAI [41] and a more plantarflexed foot posture, potentially induced by the heel lift, is associated with reduced stability and increased ankle sprain risk [42]. These results demonstrate that the custom destabilising insole can provide a targeted postural challenge for individuals with CAI, suggesting its potential as a clinically accessible tool to promote neuromuscular adaptations during weight-bearing functional exercises (standing and walking).

The observed effects of the destabilising insole are likely attributable to the combined mechanical influences of the heel lift and medial inclination on ankle joint orientation and sensorimotor demand. The heel lift places the ankle in a relatively plantarflexed posture at initial contact, a position known to reduce frontal-plane stability due to the narrow posteriorly shape of the talus [43]. Concurrently, the medial inclination created by the dual-density design biases the centre of pressure medially, generating an external inversion moment that must be countered by the peroneal musculature. In individuals with CAI, this controlled perturbation may upregulate peroneus longus activation, reflecting an adaptive increase in feedforward and feedback stabilisation strategies during early stance [10]. Collectively, the design appears to provide a task-specific stimulus that amplifies frontal-plane demands without exceeding injury thresholds, thereby acutely modulating neuromuscular control during weight-bearing tasks.

There are also some limitations in our study. Firstly, the cross-sectional design of the study only demonstrated the acute effects of the destabilisation insoles, making it difficult to elucidate whether it alters neuronuclear control strategy in the short term (after one session) or clinical outcomes in the long term (after multiple sessions). More longitudinal studies with the destabilisation insole are needed in the future. Secondly, the complex design combining with heel lift and inversion inclination made it difficult to differentiate the function role of each component. Future studies separating these components are recommended. Furthermore, the participants were not blinded to the footwear allocation due to the nature of experiment, which may introduce potential bias in the results. Lastly, other foot postures (pronated or supinated foot) of participants were not considered in current study and may limit the results applying in more general CAI population.

### Perspectives

Medial-lateral destabilisation insoles could alter neuromuscular control patterns and add additional stability challenge during walking and standing for people with CAI, which indicates the potentials to be a supplementary rehabilitation tool to restore neuromuscular function in the weighting bearing exercises. However, it should be noted that additional postural challenges including increased ankle inversion angle or accelerated reaching the stability boundary might add potential risks of re-injury during tasks, which should be minimised under carefully supervision by professionals. If any discomfort experienced by the participants during the familiarisation, health professionals should stop to use it immediately in case any further injuries. Moreover, the current study only demonstrated the acute biomechanical effects induced by the destabilisation insoles and whether those biomechanical changes could be transferred into meaningful clinical benefits in the short-term and long-term still needs further exploration.

## Conclusion

Medial-lateral destabilisation insole increased ankle inversion angle, peroneal longus amplitude during walking and decreased postural stability during standing, providing a controlled perturbation to enhance ankle neuromuscular control during gait in CAI, while simultaneously imposing a significant postural challenge during stance. These alternations indicate it as a promising, clinically accessible tool for supervised neuromuscular rehabilitation during functional weight-bearing activities, though longitudinal studies are needed to confirm long-term efficacy and safety.

## Data Availability

The datasets analysed during the current study are available from the corresponding author on reasonable request.

## Abbreviations

AP: Anterior-posterior
BF: Biceps femoris
BMI: Body mass index
CAI: Chronic ankle instability
CAIT: Cumberland ankle instability Tool
COP: Centre of pressure
COPV: Centre of pressure excursion velocity
EMG: Electromyography
FAAM ADL/SPORT: Activities of daily life/sport subscale of foot and ankle ability measure
FPI: Foot posture index
GL: Gastrocnemius lateral
GM: Gluteus medius
GRF: Ground-reaction forces
ML: Medial-lateral
MVIC: Maximum voluntary isometric contraction
PL: Peroneal longus
RF: Rectus femoris
RMS: Root mean square
TA: Tibialis anterior
TTB: Time to boundary
SPM: Statistical parametric mapping
